# Protein Biosensors Based on Polymer Nanowires, Carbon Nanotubes and Zinc Oxide Nanorods

**DOI:** 10.3390/s110505087

**Published:** 2011-05-09

**Authors:** Anish Kumar M., Soyoun Jung, Taeksoo Ji

**Affiliations:** 1 Microelectronics Photonics, University of Arkansas, Fayetteville, AR 72701, USA; E-Mail: manoanish123@gmail.com; 2 Department of Electrical Engineering, University of Arkansas, Fayetteville, AR 72701, USA; E-Mail: soyounj@yahoo.com

**Keywords:** biosensor, nanostructures, electrochemical, immobilization

## Abstract

The development of biosensors using electrochemical methods is a promising application in the field of biotechnology. High sensitivity sensors for the bio-detection of proteins have been developed using several kinds of nanomaterials. The performance of the sensors depends on the type of nanostructures with which the biomaterials interact. One dimensional (1-D) structures such as nanowires, nanotubes and nanorods are proven to have high potential for bio-applications. In this paper we review these three different kinds of nanostructures that have attracted much attention at recent times with their great performance as biosensors. Materials such as polymers, carbon and zinc oxide have been widely used for the fabrication of nanostructures because of their enhanced performance in terms of sensitivity, biocompatibility, and ease of preparation. Thus we consider polymer nanowires, carbon nanotubes and zinc oxide nanorods for discussion in this paper. We consider three stages in the development of biosensors: (a) fabrication of biomaterials into nanostructures, (b) alignment of the nanostructures and (c) immobilization of proteins. Two different methods by which the biosensors can be developed at each stage for all the three nanostructures are examined. Finally, we conclude by mentioning some of the major challenges faced by many researchers who seek to fabricate biosensors for real time applications.

## 1. Introduction

A biosensor is in general an analytical device that responds to biological detection of proteins in the form of electrical signals [1]. It can also be defined as a device in which the response of some chemical biomatrix such as antibodies, enzymes *etc.* is modified into an electrical signal depending on the concentration of the analyte used [2]. Typically, a biosensor is comprised of a transducer part and a sensing part. The detector part is the one that detects the target cells in the body and the transducer collects the information from the detector and transmits a signal to the output system. The detector part is usually a protein or an enzyme that captures the target cells, while the major part of the sensor is the transducer which changes the characteristics of the whole sensor and allows researchers to develop an effective biosensor so that it can be implanted into a human body. The structure of the transducer part is the main factor that will decide the number of available protein binding sites.

In recent times, one dimensional nanostructures such as nanowires, nanotubes and nanobelts have attracted a great attention in the construction of biosensors due to their unique properties and potential to be fabricated as sensors [3]. With a large surface/volume ratio and a Debye length comparable to the nanostructure radius, the electronic properties of these nanostructures are strongly influenced by surface processes, giving rise to superior sensitivity than their thin film counterparts. In comparison with 2-D films, where the charges are accumulated on the surface, the charge accumulation in 1-D nanostructures occurs in the bulk of the material, which ensures good electrical properties during detection. The 1-D nanostructures are most commonly fabricated by a bottom-up approach using synthesis processes. A bottom-up approach is nothing but a chemical reaction that is done using particular reactants under specific conditions. It basically requires a catalyst, a vapor phase reactant (nanostructure material) and a thermal environment to effectively synthesize the nanomaterials. These 1-D nanostructures are chosen particularly due to their high response to external stimulus that can be used for real time monitoring applications [4–11].

In this paper we review three main kinds of 1-D nanostructures, as mentioned above. The review concentrates particularly on materials such as polymers, carbon and zinc oxide (ZnO) that can be fabricated in these 1-D nanostructure forms. The materials that can be molded into these nanostructures play a key role, especially, for bio-applications. There are various methods by which these nanomaterials can be fabricated, aligned and used to immobilize proteins. Here we first discuss the materials used for fabricating nanostructures, followed by the techniques used during the three different stages of biosensor fabrication.

Conducting polymers (CPs) that possesses high electrical conductivity due to their π conjugated electrons are one of the more promising biocompatible materials and have been used in various applications [12–15]. Thus, they have been used as a transducer in biological sensors because of their attractive properties such as high stability at room temperature, good conductivity output and facile polymerization [16]. Another important advantage of using CPs is that the biomolecules can be immobilized onto the nanowire structure in a single step rather than the multiple steps that are required when other non-polymeric materials are used. In addition, the electrochemically prepared CPs can be grown with controlled thickness using lower potential and they also have an excellent enzyme-entrapping capability [17–20].

Another successful 1-D nanostructure in the field of biosensors is the carbon nanotube (CNT). These exhibit very long and slender shaped structures with high surface area, hexagonal networks, and unique C-C covalent bonding which makes them attractive in the field of biosensors [21]. The CNTs were used in the field of biosensor in order to introduce a new material than the ones that already exists. This led to the preparation of CNTs using chemical methods so that the immobilization of biomolecules could be done in a reliable manner [22]. Additionally, organic molecules integrated with nanotubes are believed to offer new research fields and applications such as *in vivo* implantation of the device [23].

Zinc oxide is a fairly recent material on which the research is being concentrated to develop it as a biosensor. ZnO had some different issues when fabricated in nanostructures than when used in a planar device. Since the nanostructures that are built using the bottom-up approach can be made to work as a biosensor system, many attempts in this direction have been made at recent times [24,25]. Properties such as the high isoelectric point (9.5) and fast electron transfer [26] observed in ZnO nanowires are also a reason for these attempts. Thus, it is believed that the usage of this material as a biosensor will also be successful in the coming years.

## 2. Biosensor Development Using Polymer Nanowires

Polypyrrole, a conducting polymer which is more conductive, stable and biocompatible than other polymer materials is reviewed in this paper. In 1916, polymerization of the monomer was first done by a research group [27] who oxidized it into a fine black amorphous powder and named it “pyrrole black”. Later, these monomers were grown by controlling the thickness of the polymer film [28], which attracted a great deal of attention from many researchers in the biomedical applications field [29]. At present, nanosize polypyrrole is grown, which allows even a very low current to pass through, making the device even compatible with the human body. According to [30], electrochemically prepared pyrroles are more conductive and stable when compared to the chemically prepared ones.

### 2.1. Synthesis of Polymer Nanowires

In 1991, Wei *et al*. followed a method which led to the production of polypyrrole powder. After this attempt, they tried to prepare the powder at an increasing rate and as a doped and conductive form using chemical polymerization techniques [30]. This kindled the notion of upgrading the material and using it in various applications. They were initially prepared using the slip casting and sol gel methods [31], involving very complex and time consuming processes, but nowadays the synthesis of the polymers is done electrochemically since the fabrication process is simple and cost effective, requiring just a porous template of some electrically insulating material. According to [32], the growth of polymer nanowires can also be done using the track edge method, printing techniques, photochemical lithography, edge lithography and micromolding [33]. However, these techniques are all very complex and require very costly equipment for the preparation of nanowires.

**Figure 1.**
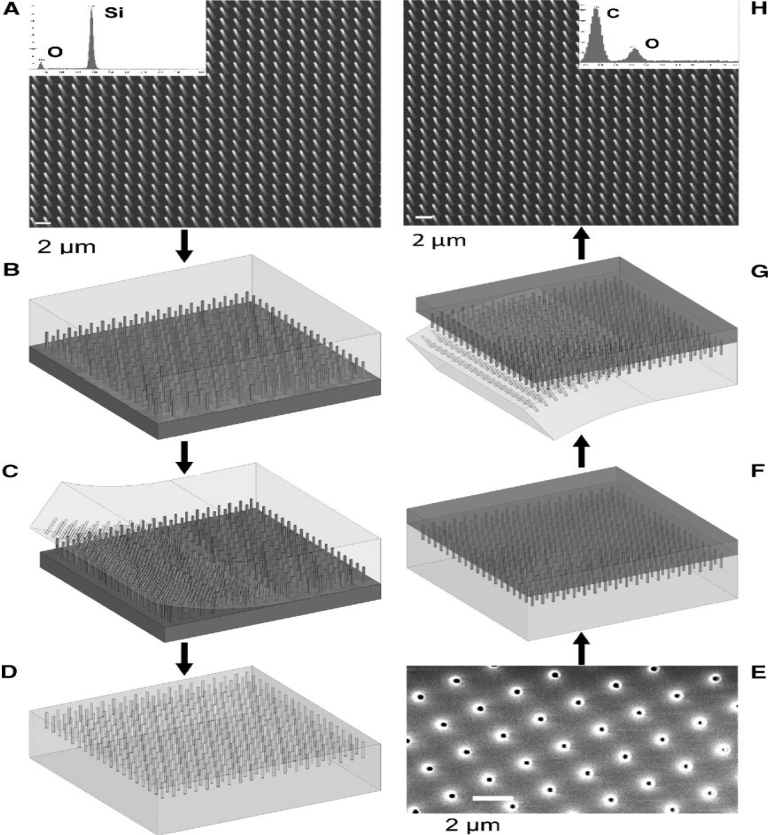
Micromolding process for fabricating polymer nanowires with high aspect ratio, **(A)** SEM image of Silicon master bearing square array posts grown using photolithography process, **(B)** Silicon master with liquid PDMS, treated with antisticking agent, **(C)** Cured PDMS peeling off from master, **(D)** Negative PDMS containing high aspect ratio hollows, **(E)** SEM image of hollow negative PDMS mould, **(F)** Polymer and liquid metal being cured in the mould, **(G)** Negative PDMS peeled off from actual polymer nanowire, **(H)** SEM image of nanostructured replica fabricated from epoxy resin (reprinted with permission from Wiley-VCH Verlag GmbH & Co. KGaA) [33]).

For instance, micromolding is one of the most effective techniques for fabricating nanowires with high aspect ratio [33]. In this method, an initial polydimethylsiloxane (PDMS) mold which is the master is produced with the desired length, radius and pitch (distance between nanowires) using a photolighraphy process. Then an antisticking layer—PDMS or paraffin—is poured into the master and cured for a few hours and later peeled off. This antisticking layer is shown in the Figure 1(D). A SEM image of the negative PDMS mold onto which the polymer solution is poured along with a supporting liquid metal such that the holes are filled up with the liquid can be seen in Figure 1(E).

Once the solution is cured, the negative PDMS mold is peeled off leaving behind the polymer nanowires. As the procedure sounds, it is a very lengthy and time consuming process. The article reports that the stability of the structures must be taken into consideration while fabricating nanoposts with high aspect ratio. Collapse of nanoposts due to their own weight, adhesion forces between adjacent nanoposts and the surface morphology needed to support the posts may cause problems, which can be solved by varying the length or the radius of the nanoposts with an appropriate selection of the supporting liquid metal.

In the track-etch method, a thin polycarbonate film was initially spin coated, irradiated with Ar^9+^ ions at 220 MeV, and UV, followed by chemical etching to finally form pores of 15 and 100 nm with lengths ranging from a few nm to μm. Polypyrrole (ppy) doped with perchlorate electrolyte was used for the synthesis of the nanowires on the track etched membrane of a nanoporous polycarbonate template [34,35]. The synthesis was done using different conditions to control the growth of the polymer layer [35]. The deposition conditions with which the polymer was completely filled the polycarbonate template up to the top was chosen as the best one, since the intention was to avoid any overgrowth of the material outside the template, as it may form like a film layer that would cause a problem during the time of immobilization of the antibody-antigen. This method of preparing polymer nanowires with certain parameters was shown to be the best method as per [36], but it has been mentioned that it is an expensive and time consuming method which involves a very complex procedure.

The preparation of alumina templates plays a major role in the synthesis of nanowires. According to one report [31], alumina templates having high thermal and electrical stability properties were prepared using an anodic oxidation process, It was mentioned that this 99.8% pure alumina membrane was prepared as a challenge to the previous issue that the alumina membranes had, whereby they displayed high pore density and uniform pore diameter, but were not able to react with water at temperatures over 80 °C without making holes through the pores and thus offering less resistance to attack by acids and alkali. They were then oxidized to form a very good porous membrane. The final membrane was ready after giving it a heat treatment and it was checked for its corrosion and chemical resistance.

The procedure for preparing the template was very lengthy and involved a lot of chemicals, even starting from the thermal oxidation of the alumina plate. Although this process is used even nowadays for preparing templates, it remains a very expensive and time consuming process, thus commercially available alumina templates are preferred as they have all the characteristics of the ones prepared in the lab.

Several methods were being used to synthesize ppy nanowires in the past, but the three electrode cell method is now widely used by many researchers due to the ease of growing nanowires and also because it is cheaper in comparison with other methods. The synthesis of ppy nanowires using a three electrode cell as reported in [16,37,38] was done using an alumina template that is commercially available on the market. They used an alumina template of 200 nm diameter and 60 μm thickness (Whatmann International). A gold layer was initially sputtered onto the back side of the template which serves as a base to the nanowire that is going to be developed. The three electrode cell method for the electrodeposition of the nanowires employed the substrate as the working electrode, platinum mesh as the counter electrode and Ag/AgCl as the reference electrode using a potentio/galvanostat. They used a mixture of 0.5 M pyrrole and 0.2 M LiClO_4_ as the electrolyte solution.

**Figure 2.**
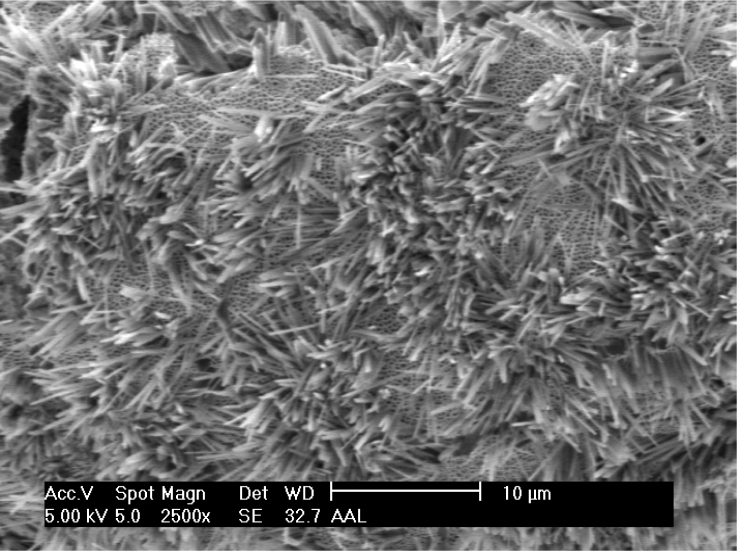
SEM images of polypyrrole nanowires grown using a three electrode system.

The whole electrodeposition process was done potentiostatically at 0.9 V for 30 min. After deposition, the gold seed layer was removed using 0.15 M KI in 0.1 M I_2_ gold etchant solution and the alumina templates were dissolved using 3 M NaOH solution. The obtained nanowires were analyzed using a SEM instrument. Figure 2 shows a SEM image of polypyrrole nanowires grown using the three electrode method. These nanowires were then suspended on the electrodes for aligning it into individual threads. This will make the system to be devised in a uniform manner and also effective for the subsequent immobilization of proteins.

### 2.2. Alignment of Nanowires

Electrochemically synthesized nanowires will be distributed randomly all over the surface of the substrate. These nanowires are then made to align in a uniform fashion so that there is no interlinking between them because the nanowires interlinked one on top of the other would lead to a situation where the proteins cannot be immobilized on the nanowires that are present below. The surface of the layer will also be non-uniform all over the device, making protein binding impossible. This would be a barrier to improving the sensitivity of any device, so the nanowires are first aligned before binding the proteins. Several methods are used for aligning nanowires among which the dielectrophoresis technique has attracted a great deal of attention due the fact that each and every nanowire can be effectively aligned. The so called mechanical break junction method was one of the methods that was earlier used for aligning polymer nanowires.

**Figure 3.**
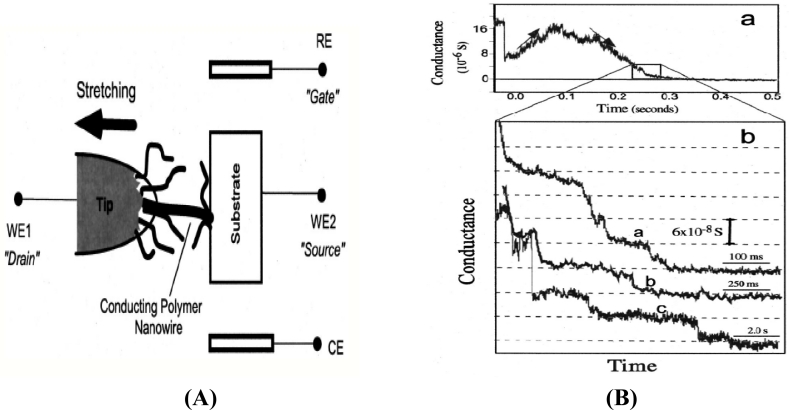
**(A)** Growth of nanowires using break junction method and **(B)** Graph showing the conductance of the polymernanowires during stretching process (reprinted with permission from the American Institute of Physics [39]).

This method works in a way that allows the material to be synthesized and aligned at the same time. The material to be polymerized (aniline, as a monomer) was first attached to a sharp tunneling microscope tip (STM) separated by an insulation layer to focus the growth of the nanowire. A mixture of 0.1 M aniline + 0.5 M Na_2_SO_4_ was used for growing the nanowire. It was first electrodeposited on the tip of an insulator at ∼1 V (*versus* Ag/AgCl reference electrode) followed by applying a potential along the tip of the substrate where the polymer starts to grow as a thin wire. It was reported that an increase in current was observed when this wire hits the substrate as seen in Figure 3, and was further stretched to reduce the thickness using a dc motor [39].

The conductance of the grown nanowire was observed using a graph plot [Figure 3(B)] and the stretching process was continued to improve the conductance of the nanowire. Although a polymer nanowire of calculated conductance can be obtained using this method, there are many drawbacks of using this method. One of these drawbacks is that only one nanowire can be grown at a time. Obviously it is desirable for fabrication processes to be faster to allow the production of multiple devices at a given particular time.

**Figure 4.**
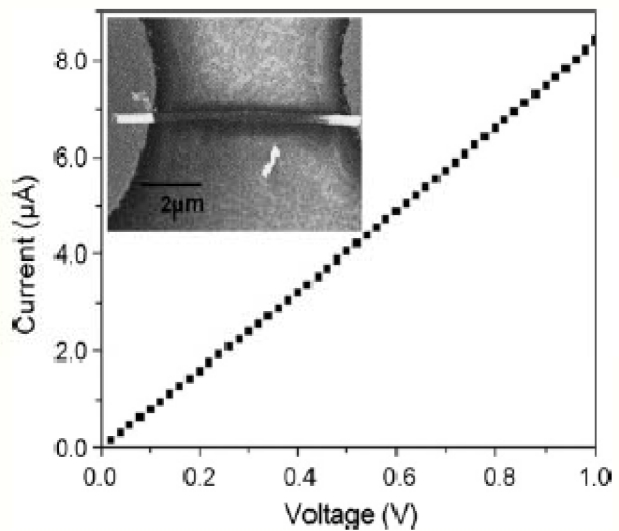
I–V characteristics of an aligned single polymer nanowire (reprinted with permission from Elsevier [44]).

The dielectrophoresis method of aligning the nanowires was used at recent times since the alignment is easier and also because comparatively, a greater amount of nanowires can be aligned and tested at the same time. First, interdigitated electrodes were fabricated on a SiO_2_ wafer using a photolithography process with chromium and gold. The polymer nanowires were then dispensed all over the electrodes in a random fashion. The presence of nanowires was verified by measuring the I-V characteristics of the electrodes, so a potential was applied across the ends of the electrodes and the current was measured with the use of a potentiostat. Figure 4 clearly shows the amount of potential applied to the electrodes making the nanowires align with a small amount of current passing through them [44]. The nanowires present in a 2 μL diluted suspension were dispensed on 16 pairs of well aligned interdigitated electrodes [40]. A 1 V potential from peak to peak and 5 MHz frequency was applied to the electrodes allowing the nanowires to align along the corresponding electric charges of the material. This means that the nanowires are present in between the electrodes with their ends touching the electrodes [37].

The electrodes act as a capacitor before the nanowires are suspended on them. The potential applied to the electrodes flows in the air medium making no electrical contact between the electrodes. These electrodes later act as a resistor after the suspension of the nanowires which makes the electrodes come in contact with each other. Usually, a probe station was used by most researchers for applying the voltage and measuring the current at the same point. After aligning the nanowires in the desired position, selective deposition of the metal was done using the three electrode cell method making an ohmic contact on the electrodes which was helpful in immobilizing the biomolecules in a easier way. The ohmic contact was also helpful in measuring the I-V characteristics. The output current depends on the number of nanowires present on the electrodes which shows that the amount of current flowing through the nanowires gets shared and the sensitivity of the sensor depends on the number of nanowires present. The next step involved in developing the biosensor is the immobilization of the biomolecules.

### 2.3. Immobilization of Proteins

The immobilization of protein constitutes the sensing part of the sensor that captures the affected cells and transmits the output in the form of an electrical signal. In 1962, Clark and Lyons [40] were the first to demonstrate a biosensor integration using an enzyme and an electrode together which had many advantages in building the sensor with high selectivity and ease of use in complex media. The immobilization was done using various methods in which the entrapment process involved fixing of proteins onto the nanowires at some constant voltage. It was later realized that the antibodies were just barely sticking onto the nanowires and were not firmly attached [41,42]. EDC crosslinking is a recent technique that involves covalent bonding between the proteins and the transducer. The immobilization in general, is said to be effective since the proteins firmly bind onto the nanowires at specific spots. The more activated sites on the nanowires and bonds to the captured proteins there are increases the sensitivity of the device.

The entrapment method of immobilizing biomolecules on the nanowire surface is well explained in [41]. According to this report, nanowires of ∼320 nm diameter and ∼2 nm long were used for the binding of proteins. Protein modified nanowires were mostly used for binding. Biotin-FITC solution in a mixture with PBS (pH 7.4) was agitated constantly at 25 °C. This mixture was then entrapped onto the nanowires by incubating the protein modified nanowires with the biotin-FITC solution. The amount of biotins attached to the nanowires was determined by a signal generated at the output. Using this method, the biomolecules were just physically entrapped onto the nanowires, which does not result in much effectiveness during sensing as a strong attachment of proteins is needed for the sensing of the antigens.

**Figure 5.**
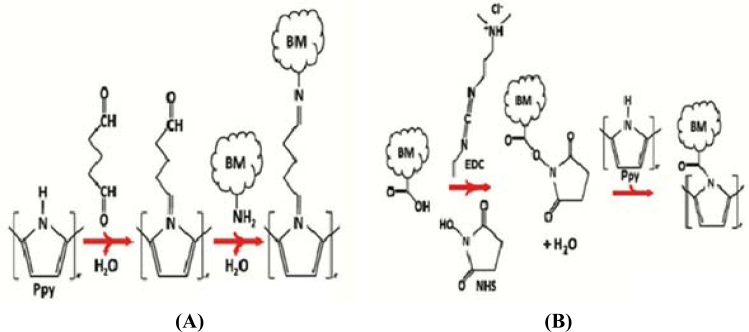
EDC cross linking showing **(A)** activation of carboxylic group in biomolecules and **(B)** activation of amine group in polypyrrole nanowires (reprinted with permission from the American Chemical Society [43]).

In [43] the EDC cross-linking technique was used, serving as the reaction layer in between the nanowire and the antibody bonding it together. A known quantity of protein was mixed with a buffer solution containing EDC and NHS, and centrifuged after agitation of the solution for 1.5 h. According to the authors the EDC/NHS will activate the carboxyl groups which are already present in the polymer nanowires, and later bond with the amine residues that are present at the ends of all the antibodies forming a strong covalent bond between the nanowires and proteins. The bonding could also be done in the reverse manner. Figure 5 shows the reactions involved in the EDC cross-linking between the nanowires and the proteins. The mixing of the buffer solution with the nanowires can either be done in a centrifuge tube [43] or also on the top of the lithographically fabricated electrodes. The presence of proteins on the nanowires was observed by the change of solution to a fluorescent color.

## 3. Electropolymerization of Carbon Nanotube Biosensor

The synthesis of carbon nanotube (CNT)-based biosensors using chemical vapor deposition is being followed at recent times due to unique properties of this material in biomedical applications [45]. It is believed that the electrochemical reactivity of the CNTs can enhance the bio-molecule binding, and also that the electron transfer reactions of proteins can be well promoted [45–48]. In 1993, Iijima and Ichihashi [49] and Bethune *et al.* [50] were the first to synthesize single walled carbon nanotubes after the discovery of CNTs in 1991. CNTs are classified into single wall (SWNTs) and multi-wall nanotubes (MWNTs). SWNT is a cylindrical shaped structure on which a layer of graphite sheet is spread and makes it appear as a tube shape, whereas, MWNTs consist of multiple graphene sheets interspaced by 3.4 Å [51,52]. Synthesis of CNTs by arc discharge evaporation, laser ablation and chemical vapor deposition are reviewed in this paper. Electrochemical detection of CNTs has also been reported using the three electrode cell method, which is believed to be a very effective process that prepares the material in such a way that the immobilization of the proteins will be excellent in comparison with the material prepared using other processes. This may also lead to production of a multi-sensing biosensor.

### 3.1. Synthesis of CNTs

In 1991, a needle like tube structure was first reported by Iijima, which was later produced using an arc discharge evaporation technique [53]. Later, the same technique was done by applying a direct current to the negative end of the carbon electrode to further grow the needle-like tube from 4 nm to 30 nm in diameter. The evaporation of carbon was done using an arc discharge chamber that had two vertical thin electrodes at the centre of the chamber filled with methane and argon gas. The lower chamber had a small dip at the lower electrode onto which the evaporation iron metal was placed and the arc discharge was applied. Argon, methane and iron were the three main constituents responsible for the synthesis of the SWNTs and MWNTs.

In 1996, Smalley and co-workers used the so-called laser ablation technique to grow SWNTs. It was developed by vaporizing graphite rods with small amounts of Ni and Co at 1,200 °C [54]. The nanotube started to grow bigger in size after this treatment. The excess particles got detached from the surface leaving out sufficient amounts of carbon that can poison the catalysis and also allowing it to terminate with a fullerene-like tip or catalyst particles.

Although high yields (>70%) of SWNTs can be obtained using these two techniques, there are some drawbacks which hinder the applications of CNTs in biosensors. These are: (a) high temperature is applied during evaporation of material onto the carbon atoms, and (b) the prepared nanotubes get tangled within the solution, making it difficult to deposit them effectively on a substrate.

**Figure 6.**
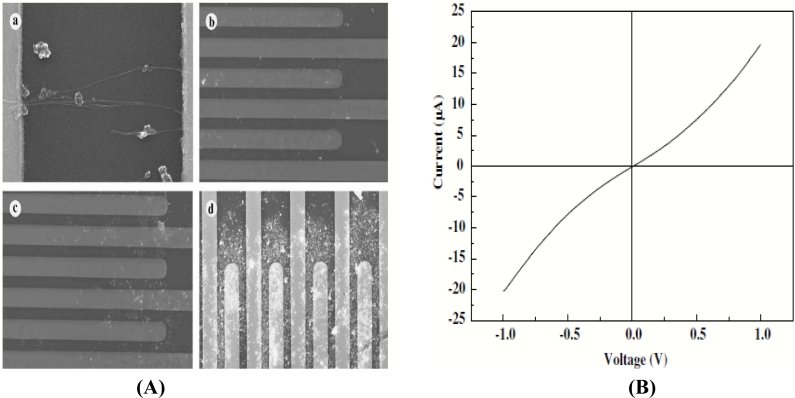
**(A)** Alignment of carbon nanotube at various voltages and **(B)** V-I characteristic of the aligned nanotubes (reprinted with permission from Elsevier [59]).

Recently, chemical vapor deposition has become the preferred technique for the synthesis of CNTs because of the two main advantages that the nanotubes can be prepared at a lower temperature and also that the prepared catalyst can be deposited on the working samples. The chemical vapor deposition was done on platinum wafers to grow the SWNTs without extraction of the oxide layer from the substrate. A mixture of 20 mg Fe(NO_3_)·9H_2_O, 5 mg MoO_2_(acac) and 15 mg alumina added to 15 mL of methanol solution was prepared. The reagents were stirred for 24 h and sonicated for 1hour and then suspended on the PMMA substrate. The substrate was heated at 170 °C for 5 min after the solvent got evaporated at room temperature. Then the CVD process was done at 1,000 °C using methane to move out the CNTs. These nanotubes were arranged on the chip for electrochemical detection [59], as shown in the Figure 6. The three electrode system was used for the detection of the nanotubes which was considered to be the most effective method that can make the CNTs highly sensitive. The setup also was used for selective binding of gold after the deposition of nanotubes. The three electrodes are the working electrode which is the CNT array, Pt wire as the counter electrode and Ag/AgCl as the reference electrode. A chamber with K_3_[Fe(CN)_6_] and amino acid as the electrolyte solution were arranged with the three electrode setup where the investigation of electrochemical characteristics of the device was successfully done.

### 3.2. Alignment of Nanotubes

We have discussed so far different methods of synthesis of nanowires, and how these nanowires are then aligned to make good contacts during the time of immobilization of the proteins. The alignment of CNTs was done using various methods such as the flow cell method [55], growth from catalyst patterns [56] and electro-spinning [57]. However, electric field assisted alignment of the nanotubes on metal electrodes is considered to be a good method since the nanotubes can be made to settle at a desired point of interest by applying an electric field. Electro-spinning and DC electric field and DC-AC electric field based methods for aligning the CNTs are reviewed in this paper.

Electro-spinning of SWNTs using the electrostatic method is one of the older techniques used for aligning them. Most researchers mix a polymer composite with CNTs for alignment and later remove it after the alignment of the structure is done. As per the report [57], poly(vinylpyrrolidone) (PVP) was doped with SWNT because of the good compatibility of the polymer with the nanotubes. This mixture was considered to be more homogenous. A thin sheet of aluminum foil was used at either ends of the fibers to drive the charge of the substrate to ground. A single piece of SWNT-PVP composite fiber was electrospun, which aligned the fiber along the aluminum ends. The PVP was later removed by heating the fiber at 600 °C, leaving the SWNT aligned. The major disadvantage of this method is that the size of fibers that are aligned decreases after decomposition of the polymer composite from the SWNT, which will increase the local charge density of the nanotubes. Another simple method is mentioned in [58] where they use micro or nanomanipulators to make an electric contact with the electrodes and move the nanotubes from one place to another for attachment. One end of the tweezer will be in contact with one of the nanotubes and the other end in contact with the other nanotube. When a voltage is applied across the nanotubes, they move closer to each other making a contact making a clamp between the tweezers.

DC field-assisted alignment of the nanotubes has been reported [59] in which the nanotubes can be formed wherever needed. According to the report, they developed an electrode having finger-like structures using photolithography and a lift off process. The alignment was performed by suspending the freshly prepared CNTs on the top of the electrode and varying the voltage for different conditions. The authors modified the nanowire density by varying the amount of nanotubes being dispersed on the electrodes. This was done by varying the concentration of the solution containing the nanotubes. I-V characteristics were used for analyzing the amount of current passing through the electrodes, as shown in Figure 6(B).

The characteristics of this process have been well analyzed and it was found that the dc electric fields increased due to the increase in density of the carbon nanotubes deposited on the electrodes. It was also observed that the ends of the carbon nanotubes came in contact with each other as they were rotating towards the applied field. This lead to combinations of many nanotubes, to become a single nanotube. It has also been reported that the nanotubes were mainly found to be present on the top of the electrodes in comparison to the nanotubes present in-between the electrodes. According to the authors, this might have occurred due to the non-uniform distribution of current to the electrodes. Liu *et al.* have proposed that the alignment of the nanotubes using an ac field [60] depends on the length of the nanowires that are being aligned in correspondence with the electrode gap that it is aligned. Their experimental results prove that longer nanotubes get aligned faster than shorter nanotubes when they are dropped in a 2 μm gap electrode and the explanation they provide for this kind of behavior is that the dielectrophoretic force will be greater on the longer nanowires in comparison to the smaller ones.

**Figure 7.**
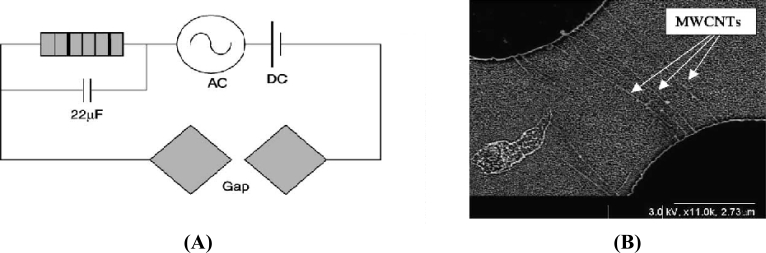
**(A)** Circuit model for single CNT deposition, **(B)** SEM image of CNT aligned using AC-DC electric field (reprinted with permission from Elsevier [61]).

Chung *et al.* have reported that by applying a combined ac and dc field [61], nanotubes can be easily aligned and deposited along the electrodes leaving behind the unwanted particles. It has been emphasized that the nanotubes respond very slowly when a dc electric field is applied, whereas the unwanted particles present in the solution respond more quickly. A schematic diagram for applying both the ac and dc electric fields has been proposed [Figure 7(A)] which has a resistor of high value in series. This resistor allows the dc potential to flow across the large resistance. There is also a capacitor present in parallel to the resistor without which a large resistance would have been resulted due to low impedance of the electrode gap. Now three different kinds of tests were performed where initially a dc voltage was applied alone to the electrodes, and it was found that the waste particles also got attracted towards the electrodes and when a pure ac voltage was applied, many nanotubes aligned together by attaching themselves to the ends of other nanotubes. The SEM image in Figure 7(B) shows the individual nanotubes that were aligned along the electrodes due to the effect of both the ac and dc voltage being applied together. A similar kind of result is published in [62], where the nanotubes are aligned using only ac, only dc and both ac and dc, but there the authors have attempted to align the nanotubes in an array fashion with single nanotubes between each gap on an electrode containing 100 such electrodes.

### 3.3. Immobilization of Antibodies

Many conventional methods are available for immobilization of proteins onto nanotubes such as enzyme linked immune-absorbent assay (ELISA) and electrophoretic immunoassay. However, since these techniques use very costly equipments to build the biosensors, electrochemical impedance spectroscopy and covalent attachment [38] of antibodies have been of much interest to effectively bind proteins. The binding of CNT using the ELISA process and covalent linking using EDC as cross-linker are reviewed. The main part of the biosensor is the attachment of the proteins to the nanowires which detects the affected cells and sends a signal as output. This part of the biosensor should be carefully devised. There are many techniques available for immobilization of the proteins onto the nanowires such as the one reported in [63,64] using three dimensional intra-molecular hydrogen bonds. A similar kind of method was followed by [65,66] in which immobilization of proteins was done by hydrogen bonding on the nanotube template by incubating both for several hours. Adsorption of antibody by non-covalent attachment onto the SWNT in a graphite disk electrode was reported by O’Connor *et al.* [67]. Kam *et al.* [68] also discussed the non-covalent attachment of biomolecules onto nanowires which they assumed to be successful by observing the enhanced attachment of poly-l-lysine (PLL) with the proteins in a hydrophobic surface of heat treated nanofibers.

The ELISA process is a complex one which consumes a lot of time just in cleaning the antibody or the proteins. It is considered to be ubiquitous in biomedical applications and clinical testing. There are two kinds of processes that can be done using the ELISA method: (a) the direct ELISA process that employs monoclonal antibodies to detect the presence of particular antigens in a sample and (b) the indirect ELISA process in which is used to determine the presence of a specific antibody in a specimen such as a serum. Huang *et al*. have reported the binding using a direct ELISA process [69]. In this process initially the antibodies are treated with water, and washed several times so that the protein surface becomes activated to bind it on the nanowires. Then the antibodies are just dispersed on the top of the carbon nanotubes for 2 h at room temperature. Later bovine serum albumin which is a blocking agent is dispersed in between the gaps of the antibodies so that the testing of the system can be done easily. After three complete washings the antigen, namely *Salmonella typhirium*, was incubated onto the antibodies for 1 h for testing the device. Finally, the binding reaction was stopped by adding 2 M NaOH solution and the binding efficiency of the antigens to the antibodies were calculated. Although the process is similar to covalent linkage during the binding process, the washing of the antigens is an added burden in the ELISA process.

**Figure 8.**
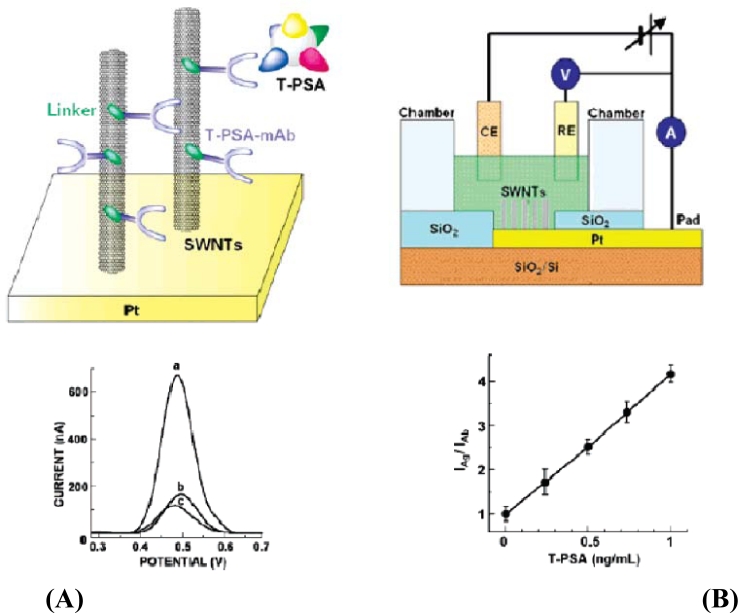
**(A) I**–V characteristics measured during the immobilization of antibodies on to the CNTs and **(B)** Schematic representation of three electrode cell for selective binding of metal for ohmic contact along with the calibration curve for T-PSA based on electrochemical signal (reprinted with permission from John Wiley & Sons [74]).

An electrochemical microelectrode containing platinum along with modified SWCNTs has been reported (Figure 8) [74] for label free detection of analytes. Electrochemical signals at various concentrations were recorded using differential pulse voltammograms. An increase in current was noticed due to the increase in concentration of the T-PSA. After many trials, it was confirmed that the addition of T-PSA onto the antibody-attached SWCNTs lead to the increase in the current. A slight defect occurred in the monolayer due to the compact packaging of the antibody on the SWCNT surface that could cause a very high electron transfer rate along the electrolyte and the CNT.

**Figure 9.**
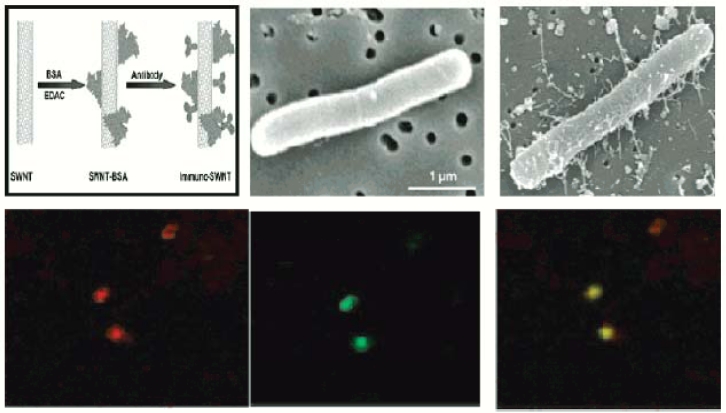
SEM images and pictures showing the presence of antibodies due to immobilization (reprinted with permission from John Wiley & Sons [69]).

The covalent bonding of antibodies provides very strong attachment to the nanotubes. There are many reports that have been discussed on the covalent bonding of proteins [70–72]. The assembled SWNTs were found laying on the electrodes ready to be immobilized. Functionalized FITC along with antibody were washed and incubated with the SWNT for several hours [73]. The conjugation between them was realized by the appearance of fluorescent color in the solution bath. Figure 9 shows the SEM images that were taken after the immobilization of the antibodies onto the carbon nanotubes. The nanotube with conjugated FITC shown in the same figure exhibits a fluorescent color due to the presence of FITC [74].

It is reported that the SWNT-immobilized protein detection is a very promising and straightforward method for use as a biosensor. Many research groups have successfully demonstrated the working of immobilized SWNT biosensor [75–77]. This kind of covalent linking using EDC is also reported in the biotin streptavidin binding [78–80]. The authors also reported that the amino group of the protein linking with the carboxyl group of the nanotubes showed a greater response than the biosensor without MWNTs. According to this, the electrochemical reaction in the MWNT-COOH would be well enhanced and would behave as a good promoter.

## 4. Electropolymerization of ZnO Biosensors

Zinc oxide is also a promising material that can be used in advanced sensor technologies due to its unique physical and chemical properties. ZnO nanorods were immobilized with protein for biosensor applications only using the FET concept. Very recently, electropolymerized ZnO nanorods for real time bio-sensing have been proposed for the first time by Liu *et al.* [81], and may be developed as biosensors for protein detection in the future. ZnO nanorods were mainly used for building solar cells [82–84], gas sensors [85], LEDs [86] and piezo-electric devices [87–89]. Although the synthesis of ZnO nanorods was done using various methods [90–92], they all require very expensive equipment. ZnO nanorods were also be synthesized using cheaper methods such as chemical bath and spray pyrolysis [93,94], but electrodeposition using the three electrode method in an AAO template is the easiest and cheapest method in comparison. The immobilization of biomolecules can also be achieved using this material.

### 4.1. Synthesis of ZnO Nanorods

Synthesis of ZnO nanorods was first reported in 1996 by Peulon *et al.* [95] who developed based on an electrodeposition method. They had observed that the oxidation needs an oxygen atom and the zinc metal cannot be deposited as it is onto the substrate. They performed the electrodeposition of ZnO nanorods using ZnCl_2_ solution with a KCl electrolyte on a glass substrate at the rate of 0.1–1 μm/h. Preparation of ZnO nanorods have also been reported by a method [96] in which the oxidation by spray pyrolysis was done using a mixture of triethanolamine and Zn (CH_3_COO)_2_·2H_2_O, and were ground with NaOH for half an hour. The so-called ZnO nanorods were then obtained by drying this chemical combination at 80 °C for 2 h. Preparation of ZnO nanorods has also been done using a quasi-spherical method in which Zn(CH_3_COO)_2_·2H_2_O was stirred with methanol at 60 °C to which drops of methanol were later added, and then dried for 24 h. These types of preparation of ZnO nanorods cannot be used for the development of flexible biosensors due to the high temperatures required for the fabrication of nanorods.

**Figure 10.**
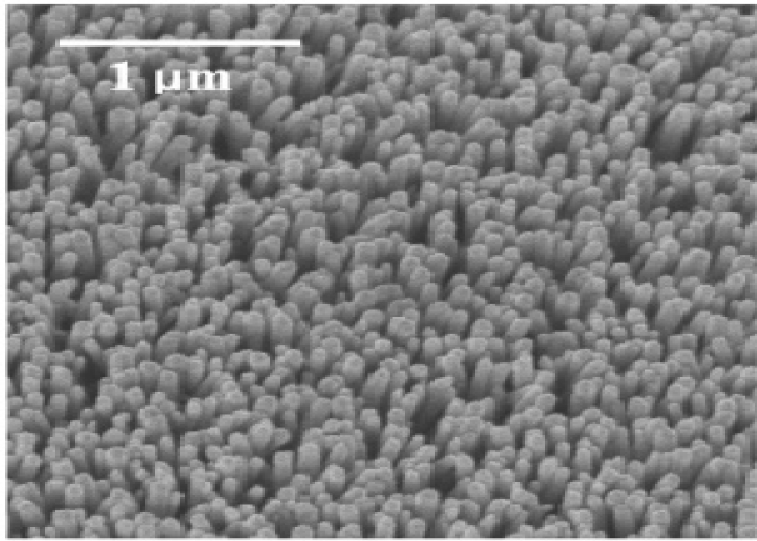
SEM image of ZnO nanorods grown on top of silicon substrate containing the seed layer (reprinted with permission from the American Chemical Society [97]).

The fabrication of ZnO nanorods at lower temperatures using aqueous solutions is also currently being done by a lot of researchers. Kumar *et al.* has proposed the growth of ZnO nanorods at a lower temperature of 95 °C [97] using zinc nitrate, Zn(NO_3_)·xH_2_O and hexamethylenetetramine for 10 h. These nanorods are actually grown on a silicon substrate having a seed layer that was coated using an ALD growth method. It is mentioned that irrespective of the seed layer charge, the nanorods will grow in an aligned manner depending on the distribution of the layer. The Figure 10 shows the SEM image of the ZnO nanorods that were grown on the silicon substrate containing the seed layer. It is also said that the ZnO nanorods will grow in an aligned manner on a sapphire surface rather than on the silicon substrate. Lee *et al.* has proven this by growing well aligned ZnO nanorods on a sapphire substrate using periodic polar inverted templates [98]. It is reported that nanorods grow well on the sapphire layer rather without any seed layer, but better growth has been reported on the silicon substrate containing the ZnO seed layer in comparison to that grown on a sapphire substrate. These kinds of aligned and patterned ZnO nanorods can also be grown using printing methods at a low temperature [99]. The nanorods grown at different heights by increasing the time period is an expected result, but it was found that the diameter of the nanorods even varied depending on the temperature at which it was grown. It is confirmed that the diameter of the nanorods increased as the solution temperature was decreased. It is also said that the diameter of the nanorods varies depending on the concentration of zinc nitrate and HMTA. This process as can be seen is very time consuming and also the sizes of the nanowires grown are very short.

Preparation of ZnO nanorods using a three electrode cell was reported by Peulon *et al.* [100]. According to the report, analysis of the mechanism of ZnO growth was done with aqueous zinc chloride solution using an electrodeposition method [101] which was a turnaround for the researchers who were working with ZnO nanorods. Electrodeposition of nanorods has been widely preferred since as mentioned for the polymer nanowires, it is an easy and cheaper fabrication method. It was shown that the use of potassium chloride (KCl) as an electrolyte solution caused a strong adsorption by the presence of anions (Cl-) that acted as a capping agent [101]. The electrodeposition was done using a three electrode arrangement containing a glass substrate as the working electrode, Pt wire as the counter electrode and saturated calomel electrode (SCE) as the reference electrode with KCl acting as an electrolyte solution. The process was done in two series in which the first series involved electrodeposition using different concentrations of 100 mL KCl electrolyte solution at 2 °C/cm^2^ charge density. In the second series, 3.4 M KCl solutions were used by varying the values of the charge density. The surface morphology was analyzed by taking scanning electron microscopy (SEM) images

**Figure 11:**
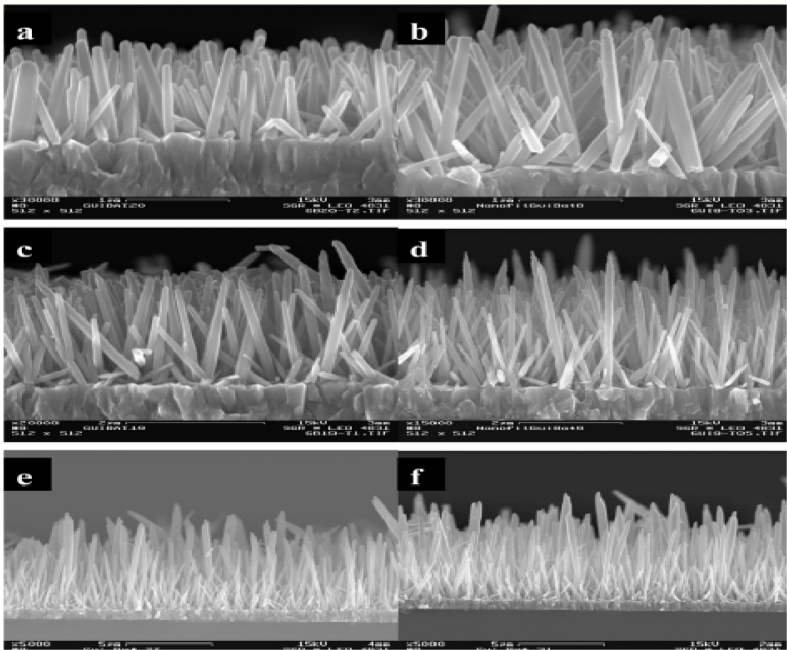
**(a–f)** Zinc oxide nanorods grown using deposition process at 90 degrees at different time periods (reprinted with permission from The American Chemical Society [94]).

ZnO nanorods for bio-sensing applications have been reported in [81], who were the first to attempt this. Synthesis of ZnO nanorods was done using a vapor solid process in which the furnace was attached to an alumina tube and a rotary pump. A polycrystalline Al_2_O_3_ boat and the purchased ZnO powder were placed inside the alumina tube which was later introduced to the furnace chamber. ZnO nanorods on polycrystalline Al_2_O_3_ was formed at a temperature of 1,400 °C with argon gas inside the chamber for 2 h. The process was repeated for different time periods to observe the effective growth of the nanowires. Figure 11(a–f) shows the SEM images of zinc oxide nanowires that were taken after the growth for different time periods. They developed a label free detection system in which amperometric sensing of analytes were done [102]. The main advantage of this report is the attachment of analytes to the nanorods, but it is a more time consuming process than the method explained above.

### 4.2. Alignment of Nanorods and Immobilization of Proteins

The synthesized nanorods were made to align along the electrodes as done for the other two materials. For the alignment of the ZnO nanorods, gold electrodes were prepared using a lift off process. The nanorods were then transferred to the Au patterned substrate using micro positioning and focused ion beam technology. They were arranged in such a way that the nanorods are in contact with the Au electrodes. Although no exact process was followed for aligning the nanorods, a micro-positioner tip of 200 nm diameter was used to separate the nanowires from each other. An ohmic contact was produced by selectively depositing a metal layer over the Au electrodes through which electric charges were applied. In the same report a similar process using a silicon substrate was also demonstrated. The analytes were integrated by injecting them with the use of a syringe and then analyzing the output. The next step after the alignment of the nanorods is the immobilization of the proteins to use as a biosensor.

**Figure 12.**
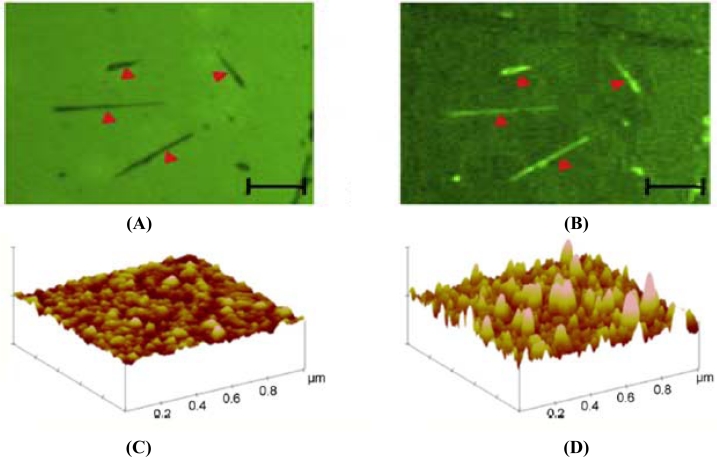
**(A)** Microscopic image and **(B)** Fluorescent microscopic image of nanowires with biotin, **(C)** AFM image of GPS+Ab and **(D)** MTS+GMBS+Ab (reprinted with permission from Elsevier [103,104]).

The immobilization of proteins onto ZnO nanorods can be effectively done by covalent attachment using either a single or a double cross-linker. For attachment via a single cross-linking layer [103], first the nanorods were immersed into a solution mixture containing ethanol with 3-(trimethoxysilyl)propyl aldehyde dissolved in water and acetic acid. The substrate containing the nanorods was treated with N_2_ gas three times at 120 °C. The antibody was activated using amine functional groups. Then the aldehyde layer present in the nanorods was made to react with the amine group-functionalized antibodies forming a very strong covalent attachment. The antibody attachment was analyzed by attaching FITC to the antibody and inspected using a fluorescence microscope. Figure 12(A,B) below show the microscopic image of nanowires with biotin and fluorescent microscopic image of nanowires with biotin, respectively. As an improvement to this method, Hunt *et al.* have done the covalent attachment of the antibodies using a double-linking layer [104]. Two samples containing the ZnO nanorods were treated with ethanol to clean the surface followed by addition of (3-glycidyloxy-propyl)trimethoxysilane (GPS) to one of the samples. The other sample was treated initially with (3-mercaptopropyl)trimethoxysilane (MTS) followed by treatment with *N*-γ-maleimidobutyryloxy-succinimide ester (GMBS) which is the second covalent linker. Finally, the antibodies were attached to both the samples which had a uniform distribution all over the surface. The activation of the layer using the cross linkers were confirmed by taking AFM images as shown in Figure 12(C,D). It is said that the fluorescence content on the GPS was comparatively less than the florescence present on the sample containing MTS, which proves that the antibody immobilization can be improved by using MTS rather than GPS.

## 5. Conclusions and Future Directions

In this paper we have reviewed different methods for the synthesis, alignment and immobilization of proteins on nanowires, nanotubes and nanorods. All three materials reviewed in this paper are widely used in the bio-sensing application field due to their promising applications. The usage of all the three materials is relatively new to this field and research using zinc oxide nanorods has only just started recently. In the future, a stable process for the identification of immobilization has to be developed. The identification using fluorescent techniques does not sound reasonable. Also the immobilization of antibodies on specific sites can be concentrated which will improve the sensitivity of the devices more. The biosensor as a whole is still facing a major challenge of implantation of the device on human as a real time device which has environmental and health issues. A lot of effort is under way to develop a device in such a way that the biosensors can be used for real time detections.
